# Exploring the link between dietary vitamin C intake and rheumatoid arthritis risk: A mendelian randomization analysis based on the National Health and Nutrition Examination Survey 2009-2018

**DOI:** 10.1097/MD.0000000000050682

**Published:** 2026-09-11

**Authors:** Ke Han, Shuqi Yuan, Suying Yan, Jiayu Fu, Tianyu Wu, Fangqin Cui, Xiaonan Zhang

**Affiliations:** a Preventive Treatment Department, Lianyungang Hospital of Jiangsu Province Hospital of Traditional Chinese Medicine, Lianyungang, China; b Department of Rheumatology and Immunology, The First Affiliated Hospital of Bengbu Medical University, Bengbu, China; c Department of Preventive Medicine, Bengbu Medical University, Bengbu, China; d Department of Pathophysiology, Bengbu Medical University, Bengbu, China.

**Keywords:** mendelian randomization, NHANES, plasma proteins, rheumatoid arthritis, vitamin C

## Abstract

Prior studies on dietary vitamin C and rheumatoid arthritis (RA) risk are inconsistent and confounded by lifestyle and reverse causation, leaving causality unclear. To address this knowledge gap, this study aimed to investigate whether higher dietary vitamin C intake is associated with lower RA risk and whether this association reflects a causal protective effect. Given that vitamin C deficiency is common in patients with RA, this study analyzed data from 49,693 participants in 2009 to 2018 National Health and Nutrition Examination Survey (NHANES) database to investigate the association between vitamin C intake and the disease. The National Health and Nutrition Examination Survey is the only U.S. national survey to provide clinical, laboratory, and nutritional data for participants of all ages. The participants were divided into quartiles based on interquartile ranges. With adjustment for all covariates, the Q3 (106.71 ± 24.81 mg/d) odds ratio = 0.937, 95% confidence interval (CI): 0.936–0.938, *P* < .001) and Q4 groups (560.61 ± 353.41 mg/day) odds ratio = 0.940, 95% *CI*: 0.939–0.942, *P* < .001) presented significantly lower risks of RA occurrence compared with the Q1 group (20.47 ± 11.99 mg/day). To overcome confounding and assess causality, we employed 2-sample mendelian randomization (MR) using genetic variants as instrumental variables. The inverse variance weighted method with MR revealed no significant relationship between RA and vitamin C (*OR* = 0.932, 95% *CI*: 0.596–1.458, *P* = .796). After adjusting for covariates (age, educational level, and medical history), the risk of RA in women was found to be significantly higher than that in men (*OR* = 1.534, 95% *CI*: 1.527–1.531, *P* < .001). However, after further adjustment for vitamin C intake, this risk showed a slight reduction (*OR* = 1.529, 95% *CI*: 1.527–1.531, *P* < .001). Two-sample MR revealed 24 proteins associated with RA. The findings on target intersection suggested the possible influence of vitamin C on RA through retinol dehydrogenase 16 and acid phosphatase 1though their synovial tissue expression (GSE55235) showed no significant differences in RA patients. This study provides observational evidence linking higher dietary vitamin C intake to lower RA risk, but MR found no causal relationship, suggesting confounding.

## 1. Introduction

Rheumatoid arthritis (RA) represents an autoimmune disease mainly featuring a persistent synovial inflammation; this condition predominantly affects women and the elderly.^[1]^ In the late-stage RA, patients also often experience extra-articular multisystem inflammation, psychological disorders, or disability, which result in the substantial impairment of the quality of life.^[2]^ Dietary nutritional interventions have gained attention as a means to decreasing the risk of chronic inflammatory diseases.^[3]^ Various dietary patterns can decrease the disease activity in RA via anti-inflammatory activities, inhibition of oxidative stress, or regulation of the balance of gut microbiota.^[4,5]^ The exogenous nutrient vitamin C (L-ascorbic acid) can be found in abundant levels in fruits and vegetables with antioxidant, immunomodulatory, anti-inflammatory, and collagen-promoting production function.^[6–8]^ Vitamin C dietary intake has shown potential in reducing the risk of chronic autoimmune diseases.^[9–13]^ Clinical trials have demonstrated the prevalence of vitamin C deficiency in RA patients often.^[14]^ A significant correlation has also been demonstrated between RA and serum vitamin C levels, indicating that a high serum vitamin C level may act as a protective factor against RA.^[15]^ However, studies hardly investigated the association between dietary vitamin C intake and RA.

Given the inherent limitations of observational designs such as susceptibility to confounding and reverse causation definitive causal conclusions cannot be drawn. To address this gap, we employed a 2-pronged approach: a large-scale cross-sectional analysis of the 2009 to 2018 National Health and Nutrition Examination Survey (NHANES) database to quantify the association between dietary vitamin C intake and instrumental variables RA prevalence, and a 2-sample Mendelian randomization (MR) analysis using genetic variants as to assess causality free from confounding.

The NHANES database contains extensive information on population health and nutrition.^[16]^ This research included the data on 49,693 participants from the NHANES 2009 to 2018, which initially explored the relationship between various levels of vitamin C intake and the incidence risk of RA. Given the inherent limitations of observational study designs, such as susceptibility to confounding factors and reverse causation, which preclude definitive causal conclusions, the study employed a 2-sample MR approach to further investigate the association between vitamin C and RA. Meanwhile, screening of RA-associated plasma proteins was conducted based on MR. The obtained intersection of vitamin C targets with RA-associated plasma proteins enabled the exploration of vitamin C and its target plasma proteins when influencing RA.

The primary objective of this study was to determine whether higher dietary vitamin C intake is causally associated with reduced RA risk. By triangulating observational and genetic evidence, this study aims to clarify the nature of the relationship between dietary vitamin C intake and RA risk, thereby informing future preventive strategies.

## 2. Methods

### 2.1. Design

The NHANES 2009 to 2018 cohorts were used to obtain demographic, dietary, and questionnaire data. NHANES comprises a large-scale epidemiological dataset, which includes an extensive sample size and high reliability of results, constructed via the National Center for Health Statistics. NHANES received ethical approval from the National Center for Health Statistics Ethics Review Board, and all participants gave informed consent before joining the study.^[17]^

**Figure 1.**
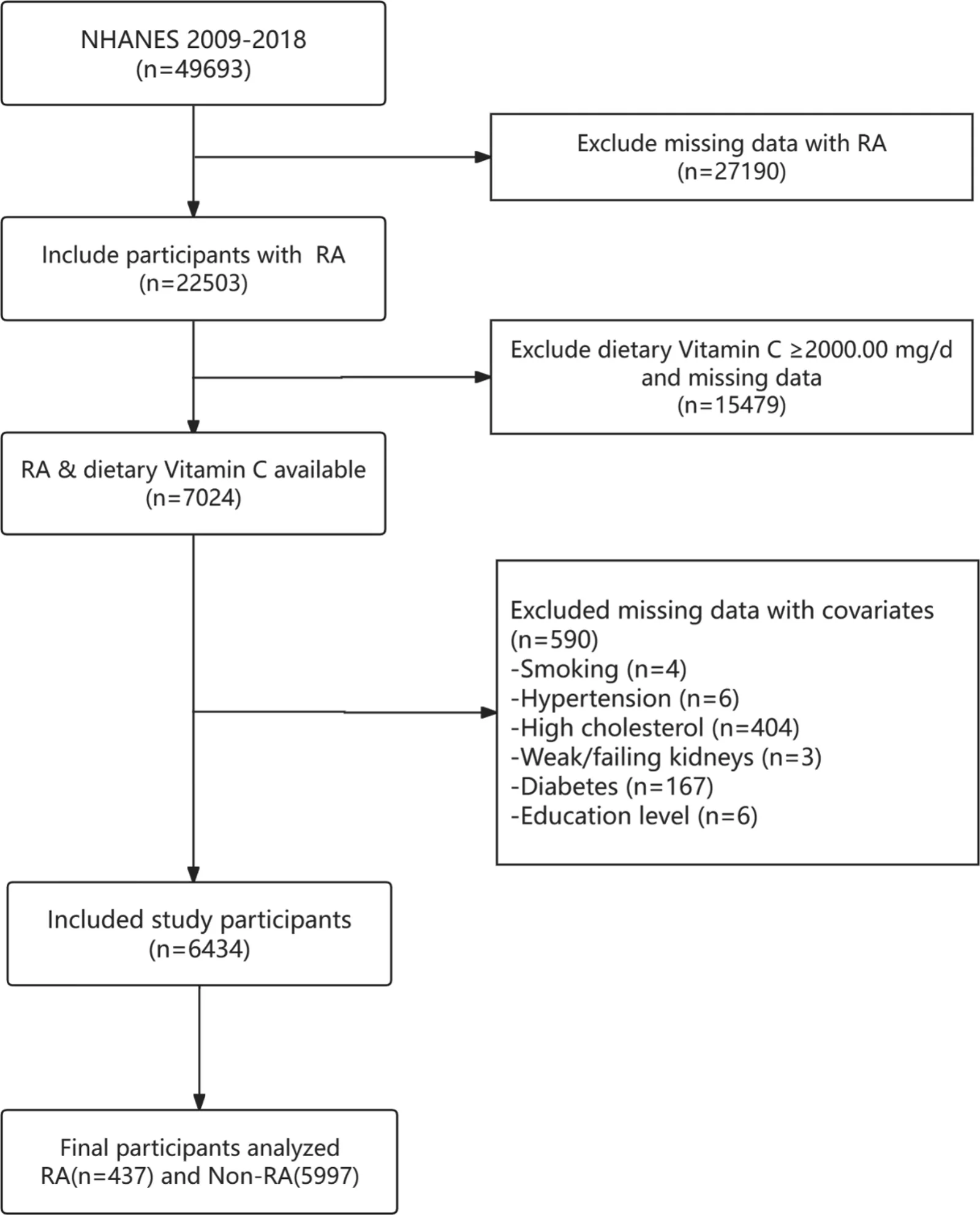
Flowchart for inclusion and exclusion of participants. RA = rheumatoid arthritis, NHANES = National Health and Nutrition Examination Survey.

A total of 49,693 participants were drawn from the NHANES database from 2009 to 2018. The inclusion criteria were as follows: Individuals diagnosed with RA and non-RA participants, defined as those without a self-reported diagnosis of RA or any other rheumatic disease, were included in the study; dietary vitamin C intake below 2000.00 mg/day without missing data and no missing data on smoking, hypertension, high cholesterol, weak/failing kidneys, and diabetes. In accordance with these criteria, final analysis included 6434 participants, comprising 437 RA and 5997 non-RA patients (Fig. 1).

### 2.2. Exposure factors and covariates

According to the World Health Organization, European Food Safety Authority, and the U.S. Food and Drug Administration the recommended daily intake of vitamin C supplementation should not exceed 2000 mg.^[17]^ Higher doses of vitamin C supplementation may lead to adverse effects, including gastrointestinal reactions. Therefore, the samples whose dietary vitamin C intake exceed 2000.00 mg/day were disregarded. Estimation of dietary vitamin C intake was completed using individual interview data from 30 days of dietary supplement use. The interquartile range method was used to categorize vitamin C intake into 4 groups: Q1 (0–40.00 mg/day), Q2 (40.01–60.00 mg/day), Q3 (60.01–166.67 mg/day), and Q4 (≥166.68 mg/day). Covariates included gender, age, race/ethnicity, education, smoking, hypertension, high cholesterol, weak/failing kidneys, and diabetes based on literature references.^[18,19]^

### 2.3. MR analysis of vitamin C and RA

MR analysis was conducted using genetic data from the Finngen and UK Biobank databases. Summary statistics were used for RA (finngen_R10_M13_RHEUMA) and vitamin C (ukb-b-19390). The instrumental variables included single-nucleotide polymorphisms that exhibited a strong association with vitamin C (*P* < 5e–06). SNPs were pruned for linkage disequilibrium (10,000 kb window, *r*^2^ < 0.001) to ensure the independence of IVs and eliminate linkage disequilibrium. The exclusion of weak instruments (*F* < 10) resulted in 10 SNPs for further analysis. Two-sample MR 0.4.6 package was used to explore the causal association via MR-Egger, weighted median, inverse variance weighted (IVW), simple mode, and weighted mode methods.

### 2.4. MR analysis of plasma proteins and RA

The data on plasma proteins were obtained from publicly available datasets.^[20]^ The selected IVs included SNPs with a strong association with plasma proteins (*P* < 5e–08). The same screening criteria and analytical methods were applied as described above.

### 2.5. Vitamin C target collection and intersection analysis of RA-associated plasma proteins

Vitamin C target data were sourced from The Comparative Toxicogenomics Database (https://ctdbase.org/), SwissTargetPrediction (http://swisstargetprediction.ch/), Binding Database (https://www.bindingdb.org/),TargetNet(http://targetnet.scbdd.com/), and PharmMapper (https://lilab-ecust.cn/pharmmapper/) databases.^[21]^

The selection standard for TargetNet was area under the curve ≥ 0.7; the selection standard for PharmMapper was a normalized fit score ≥ 0.1. Default parameters were used for the other databases. Target consolidation and deduplication were achieved using R 4.3.2 to obtain the final vitamin C targets. The identified RA-associated plasma proteins and vitamin C targets were intersected. The expression of screened plasma proteins were explored using the GSE55235 dataset from the Gene Expression Omnibus database.

### 2.6. Statistical analysis methods

Data analysis was performed through SPSS 25.0 and R 4.3.2. Continuous variables were shown as (mean ± standard deviation) and categorical variables as frequency (%). Between-group differences were evaluated via a Chi-square test. Weighted multivariable logistic regression enabled the exploration of the effect of vitamin C on RA. Three models were developed to assess the independent relationship between dietary vitamin C intake and RA: crude model (without covariates), model 1 (adjusted for gender, age, education, and race/ethnicity), and model 2 (additionally adjusted for smoking, hypertension, high cholesterol, weak/failing kidneys, and diabetes). Examination of the relationship between dietary vitamin C intake and survival outcomes was achieved through univariate and multivariate unweighted Cox regression analyses. The threshold for statistical significance was established at *P* < .05.

## 3. Results

### 3.1. Baseline of participants

A total of 6434 participants, including 437 with RA (6.8%) and 5997 non-RA (93.2%) patients, were enrolled in the study. The overall population had an average vitamin C intake of (184.74 ± 281.70) mg/day. The participants were allotted to 4 groups based on the levels of their vitamin C intake: Q1 (20.47 ± 11.99 mg/day), Q2 (58.06 ± 4.53 mg/day), Q3 (106.71 ± 24.81 mg/day), and Q4 (560.61 ± 353.41 mg/day).

The results of Chi-square test (Table 1) indicated significant differences in dietary vitamin C intake in terms of the age, gender, race/ethnicity, education, smoking, hypertension, high cholesterol, diabetes, and RA. Meanwhile, no significant difference was observed between vitamin C intake levels and a history of weak/failing kidney function, although insufficient plasma vitamin C is common in chronic kidney disease due to dialysis clearance and increased utilization of vitamin C from oxidative stress.^[22]^ (*P* = .063).

**Table 1:** Demographic characteristics of included study participants.

	Vitamin C (mg/d)					
	Q1	Q2	Q3	Q4	All	*P* value
	(20.47 ± 11.99)	(58.06 ± 4.53)	(106.71 ± 24.81)	(560.61 ± 353.41)		
**Age, n(%**)						<.001
20-39 yr	760 (45.4)	463 (26.9)	506 (35.3)	441 (27.4)	2170 (33.7)	
40-59 yr	619 (37.0)	617 (35.9)	498 (34.7)	525 (32.6)	2259 (35.1)	
≥60 yr	294 (17.6)	638 (37.1)	431 (30.0)	642 (39.9)	2005 (31.2)	
Gender, n(%)						.002
Male	702 (42.0)	795 (46.3)	661 (46.1)	778 (48.4)	2936 (45.6)	
Female	971 (58.0)	923 (53.7)	774 (53.9)	830 (51.6)	3498 (54.4)	
Race/ethnicity, n(%)						<.001
Mexican American	235 (14.0)	176 (10.2)	180 (12.5)	153 (9.5)	744 (11.6)	
Other Hispanic	161 (9.6)	126 (7.3)	140 (9.8)	155 (9.6)	582 (9.0)	
Non-Hispanic White	592 (35.4)	773 (45.0)	618 (43.1)	765 (47.6)	2748 (42.7)	
Non-Hispanic Black	331 (19.8)	342 (19.9)	240 (16.7)	267 (16.6)	1180 (18.3)	
Other Race	354 (21.2)	301 (17.5)	257 (17.9)	268 (16.7)	1180 (18.3)	
Education, n(%)						.007
High school or under	533 (31.9)	621 (29.2)	480 (33.4)	496 (30.8)	2001 (63.4)	
High school above	1140 (68.1)	1097 (63.9)	955 (66.6)	1112 (69.2)	4104 (63.4)	
Hypertension, n(%)						<.001
Yes	399 (23.8)	659 (38.4)	427 (29.8)	588 (36.6)	2073 (32.2)	
No	1274 (76.2)	1059 (61.6)	1008 (70.2)	1020 (63.4)	4361 (67.8)	
Smoking, n(%)						<.001
Yes	541 (32.3)	671 (39.1)	541 (37.7)	614 (38.2)	2367 (36.8)	
No	1132 (67.7)	1047 (60.9)	894 (62.3)	994 (61.8)	4057 (63.2)	
High cholesterol, n (%)						
Yes	464 (27.7)	623 (36.6)	499 (34.8)	577 (35.9)	2163 (33.6)	
No	1209 (72.3)	1095 (63.7)	936 (65.2)	1031 (24.1)	4271 (66.4)	
Diabetes, n(%)						<.001
Yes	136 (8.1)	244 (14.2)	151 (20.7)	200 (12.4)	731 (11.4)	
No	1537 (27.0)	1474 (85.8)	1284 (89.5)	1048 (87.6)	5703 (88.6)	
Weak/failing kidneys, n(%)						.063
Yes	36 (2.2)	60 (3.5)	32 (2.2)	43 (24.4)	171 (2.7)	
No	1637 (97.8)	1658 (96.5)	1403 (97.8)	1565 (97.3)	6263 (97.3)	
Arthritis, n(%)						.002
RA	88 (5.3)	144 (8.4)	87 (6.1)	118 (7.3)	437 (6.8)	
Non-RA	1585 (94.7)	1574 (91.6)	1348 (93.9)	1490 (92.7)	5997 (93.2)	

### 3.2. Association between Vitamin C intake and RA

Three models were established to scrutinize the association between vitamin C intake levels and RA (Table 2). The crude model revealed a certain association between vitamin C intake and low risk of RA incidence: Q2 (OR = 0.704, 95% confidence interval (CI): 0.703 to 0.705, *P* < .001), Q3 (*OR* = 0.955, 95%*CI*: 0.954–0.956, *P* < .001), and Q4 (*OR* = 0.752, 95%*CI*: 0.751–0.753, *P* < .001). Models 1 and 2 results unveiled an association between high-dose vitamin C intake and a low risk of RA incidence. However, Models 1 and 2 denoted the possibly increased risk of RA incidence with vitamin C intake Q2 (58.06 ± 4.53 mg/day) group.

**Table 2:** Weighted multivariate logistic analysis of the association between Vitamin C intakes and rheumatoid arthritis.

	Crude model		Model 1		Model 2	
	*OR* (95%*CI*)	*P* value	*OR* (95%*CI*)	*P* value	*OR* (95%CI)	*P* value
Q1	Reference	Reference	Reference	Reference	Reference	Reference
Q2	0.704 (0.703–0.705)	<.001	1.085 (1.084–1.087)	<.001	1.129 (1.127–1.130)	<.001
Q3	0.955 (0.954–0.956)	<.001	0.981 (0.980–0.982)	<.001	0.937 (0.936–0.938)	<.001
Q4	0.752 (0.751–0.753)	<.001	0.926 (0.924–0.927)	<.001	0.940 (0.939–0.942)	<.001

Crude model.

Model 1:Gender, age, education, race.

Model 2: Model 1 + hypertension, diabetes, smoking, high cholesterol, weak/failing kidneys.

As female transition into middle and old age, changes in estrogen levels contribute to an increased incidence of RA, highlighting the significant role of sex differences in RA risk. In this study, we also explored the relationship between vitamin C intake and the risk of RA in female (Table 3). Consistent with existing findings, our unadjusted model showed that compared to male, female had a significantly higher risk of developing RA (*OR* = 1.200, 95% *CI*: 1.218–1.221, *P* < .001). After adjusting for covariates such as age, educational level, and medical history, the association remained significant, with female exhibiting an even higher risk of RA compared to male (*OR* = 1.534, 95% *CI*: 1.527 to 1.531, *P* < .001). However, when vitamin C intake was incorporated into the mode l, we observed a slight reduction in RA risk among female compared to Model 1 (*OR* = 1.529, 95% *CI*: 1.527–1.531, *P* < .001).

**Table 3:** The relationship between vitamin C intake and the risk of RA in female.

Characteristics	Crude model		Model 1		Model 2	
	*OR* (95%*CI*)	*P* value	*OR* (95%*CI*)	*P* value	*OR*(95%*CI*)	*P* value
Male	Reference	Reference	Reference	Reference	Reference	Reference
Female	1.200 (1.218–1.221)	<.001	1.534 (1.532-1.536)	<.001	1.529 (1.527-1.531)	<.001

Crude model.

Model 1: Gender, age, education, race, hypertension, diabetes, smoking, high cholesterol, weak/failing kidneys.

Model 2: Model 1 + vitamin C intake.

### 3.3. Association between vitamin C intake and adverse outcomes

**Figure 2.**
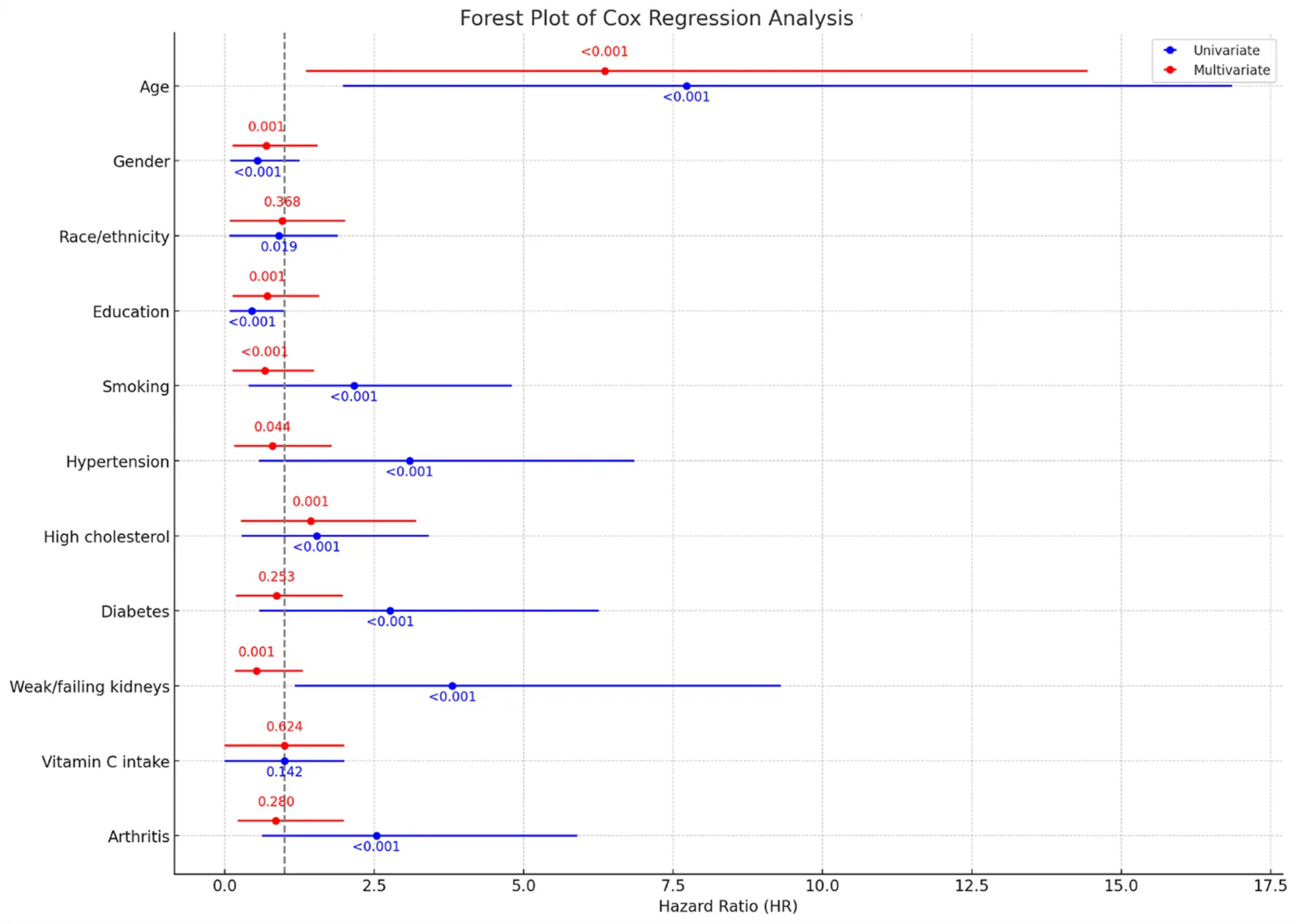
Cox regression explores the relationship between vitamin C and mortality outcomes.

According to Cox regression analysis (Fig. 2), age, gender, race, education, and medical history all showed a significant association with mortality outcomes (*P* < .05). Nonetheless, dietary vitamin C intake and mortality outcomes showed no notable association (hazard ratio (HR) = 1.000, 95% *CI*: 1.000–1.001, *P* = .142). To determine the possible fluence of covariates on the result, we performed multivariate unweighted Cox regression for further analysis of the association between vitamin C dietary levels and mortality outcomes. The findings also revealed no significant association between vitamin C intake and mortality outcomes (*HR* = 1.000, *P* = .280).

### 3.4. MR analysis exploring the causal association between vitamin C and RA

From a genetic perspective, MR was performed to investigate the causal relationship between vitamin C and RA, with vitamin C and RA as the exposure and outcome variables, respectively. The findings of MR–Egger (*P* = .845) and IVW (*P* = .759) methods suggest the lack of a causal association between vitamin C and RA (Table 4). Despite the heterogeneity observed in MR analysis (Supplementary Fig 1, Supplemental Digital Content 1), the assessment was mainly focused on the relationship between variables via the random-effects model IVW, with such heterogeneities exhibiting a slight influence on analytical outcomes. Therefore, the causal association between genetic variation and the exposure and outcome variables was non significant. This indicates that there is only an association between vitamin C and RA, but a causal relationship cannot be established.

**Table 4:** Causal relationship between vitamin C and RA.

Exposure	nSNP	Method	*P* value	*OR* (95%*CI*)
Vitamin C	10	Mendelian randomization Egger	.845	0.811 (0.257–3.019)
	10	Weighted median	.184	1.320 (0.876–1.989)
	10	Inverde variance weighted	.759	0.932 (0.596–1.458)
	10	Simple mode	.307	1.347 (0.785–2.311)
	10	Weighted mode	.134	1.423 (0.935–2.165)

### 3.5. Association between vitamin C and plasma proteins in RA

Although MR results reveal the lack of significant association between dietary vitamin C intake and RA, epidemiological studies implied the decreased risk of RA occurrence with high-dose vitamin C intake. The absorption of vitamin C, which is distributed throughout the body as a water-soluble vitamin, occurs through the gastrointestinal tract.^[23]^ Thus, we further investigated the relationship of relevant targets of vitamin C with RA-related plasma proteins.

**Figure 3.**
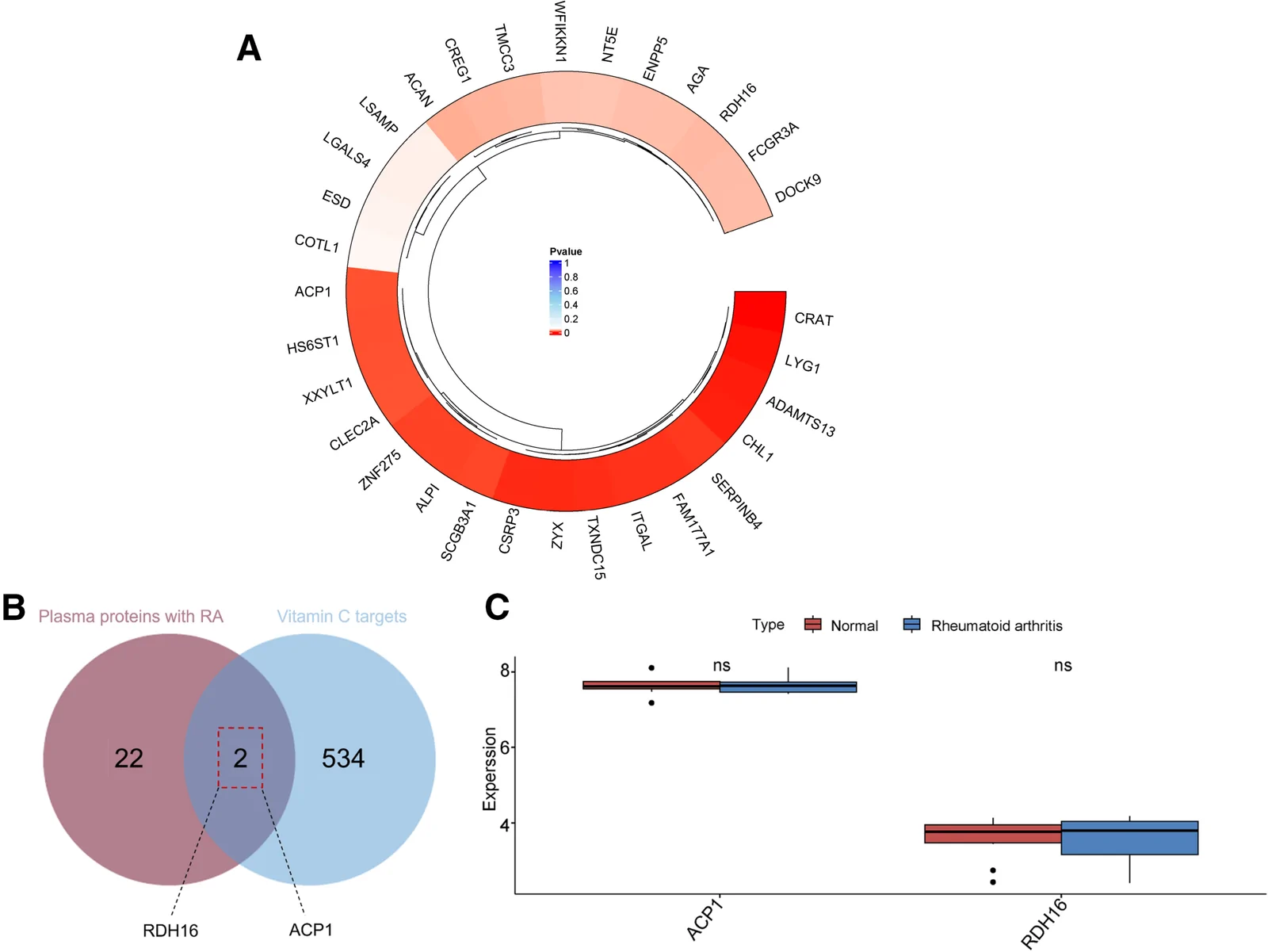
Vitamin C-RA related plasma protein screening. (A): Plasma proteins associated with RA screened by MR method. (B): Intersection of vitamin C with RA-associated plasma proteins; (C): Expression of RDH16 and ACP1 in synovial tissues of RA patients. ACP1 = acid phosphatase1, MR = Mendelian randomization, RA = rheumatoid arthritis, RDH16 = retinol dehydrogenase 16.

Based on the publicly available dataset comprising 4907 plasma proteins obtained from the work of Ferkingstad et al 24 plasma proteins potentially implicated in RA were screened via MR (Fig 3A and Table 5). In addition, 552 potential targets of vitamin C were identified using online databases. The findings indicate that only retinol dehydrogenase 16 (RDH16) and acid phosphatase 1 (ACP1) intersected between vitamin C and RA-associated plasma proteins (Fig. 3B). This outcome suggests that the action of vitamin C on peripheral blood proteins may not be the primary mechanism influencing RA. In addition, differential analysis findings suggested that RDH16 and ACP1 showed no differential expression in RA main effector tissues (Fig. 3C).

**Table 5:** Mendelian randomization study predicted plasma protein with RA.

Exposure	nSNP	Method	*P* value	*OR* (95%*CI*)
CRAT	3	Inverse variance weighted	.011	1.683 (1.293–2.191)
LYG1	3		.031	1.271 (1.108–1.458)
ITGAL	3		.037	0.711 (0.566–0.893)
TXNDC15	21		.040	0.918 (0.867–0.971)
CHL1	11		.041	0.884 (0.819–0.955)
FAM177A1	15		.041	0.917 (0.865–0.972)
CSRP3	3		.042	1.589 (1.177–2.147)
SERPINB4	6		.043	0.819 (0.714–0.939)
ADAMTS13	20		.044	1.094 (1.036–1.156)
CLEC2A	6		.044	0.866 (0.779–0.964)
XXYLT1	4		.045	0.866 (0.779–0.964)
ACP1	9		.046	0.944 (0.905–0.985)
ZNF275	3		.047	1.463 (1.117–1.915)
ZYX	4		.048	1.500 (1.154–1.948)
SCGB3A1	31		.048	1.083 (0.697–0.985)
AGA	4		.048	0.828 (0.806–0.968)
HS6ST1	15		.048	0.883 (0.697–0.985)
DOCK9	3		.049	1.299 (1.022–1.652)
ENPP5	19		.049	1.064 (1.005–1.126)
NT5E	18		.049	1.077 (1.003–1.080)
FCGR3A	16		.049	1.041 (1.036–1.156)
WFIKKN1	4		.049	1.166 (1.011–1.345)
RDH16	4		.049	1.192 (1.015–1.400)
LSAMP	12		.049	0.890 (0.795–0.998)

ACP1 = ac**i**d phosphatase 1, RDH16 = retinol dehydrogenase 16.

## 4. Discussion

Scholars have diverted their attention to the effect of balanced diet and the use of dietary supplements on the development of chronic diseases.^[24]^ RA is a multifactorial mediated inflammatory disease. Alterations in the risk of RA or disease activity through multiple pathways can be attributed to food composition, traits, and dietary patterns as controllable environmental risk factors.^[25–27]^ A high intake of red meat, saturated fatty acid, trans fatty acid, and highly refined carbohydrates can increase the risk of RA development.^[28]^ This condition may probably be due to the obesity associated with western-like dietary patterns, which result in the increased proportion of white adipose and promote the expressions of interleukin 6, C-reactive protein, and other pro inflammatory factors.^[29,30]^ Different from pro-inflammatory dietary patterns, plant-based or anti-inflammatory diet patterns can reduce RA disease activity. Philippou E et al conducted a meta-analysis and suggested that that long-term consumption of omega-3 polyunsaturated fatty acids can reduce disease activity and alleviate medication failure in RA.^[31]^ Other studies showed that a high consumption of plant foods rich in polyphenols, alkaloids, and other compounds positively affects the prevention and supportive treatment of RA.^[32,33]^ Vitamin C is a popular dietary supplement diet due to its low cost, remarkable antioxidant properties, and immunomodulatory effects. Vitamin C as a dietary supplement plays crucial roles in diseases, such as pneumonia,^[34,35]^ sepsis,^[36]^ and diabetes.^[12]^ It has been confirmed that ascorbic acid possesses the ability to inhibit the generation of self-reactive plasma cells, reduce the production of autoantibodies, and thereby delay the onset of RA.^[37]^ Zhao et al observed the higher levels of *E. coli* and *S. bovis* in the early gut environment of RA patients compared with those in later-stage RA and osteoarthritis patients and a direct correlation between the relative abundance of *E. coli* and *B*. *bovis* and the levels of tumor necrosis factor and interleukin 6.^[38]^ Cheng M et al demonstrated a strong correlation between elevated levels of *E. coli* and rheumatoid factor in the early and middle stages of RA.^[39]^ However, the relative abundances of *E. coli* and *S. bovis* decreased during RA progression. In addition, *E. coli* and *S. bovis* promoted the degradation of vitamin C, which contributed to the progression of RA.^[40,41]^ Hence, the low doses of vitamin C did not substantially reduce the risk of disease development because *E. coli*, *S. bovis*, and other gut microbiota can degrade low doses of vitamin C and may produce degradation products that boost inflammatory responses and increase disease activity in RA via the gut–joint axis. The same findings on the cohort study by Cerhan JR et al suggest that low-dose vitamin C supplementation may be unrelated to the risk association for the development of RA.^[42]^ However, the current correlation between vitamin C consumption and RA remains ambiguous.

The epidemiological data from the NHANES database in 2009 to 2018 indicated that various doses of vitamin C intake can reduce the risk of RA occurrence in the crude model constructed via weighted logistic regression. Compared with a low-dose intake, vitamin C intake levels above 60 mg/day may contribute to the reduced risk of RA occurrence. However, no substantial correlation was detected between dietary intake of vitamin C and adverse composite outcomes. Notably, vitamin C intake elevated the risk of RA occurrence within the range of (58.06 ± 4.53) mg/day, which is contrary to conventional knowledge. Although the cross-sectional analysis of the NHANES data suggested an inverse association between high-dose dietary vitamin C intake within tolerable upper intake levels and the prevalence of RA, these findings are observational and cannot establish causality.

Male exhibit a significantly lower risk of autoimmune rheumatic diseases compared to female. This is attributed to the direct and indirect anti-inflammatory effects of androgens on the immune system, whereas estrogens exert more complex pro-inflammatory and anti-inflammatory roles, likely dependent on the cell type targeted (e.g., T or B cells), dosage, concentration, and other factors.^[39]^ When vitamin C intake was included as a variable in the analysis, a slight reduction in RA risk was observed among women.

Most of the current research on the treatment of RA with vitamin C are based on the high-dose vitamin C shock therapy conducted in animal experiments. Randomized controlled trials demonstrating that vitamin C intake below the UL can promote the prevention or treatment of RA are still lacking. Vitamin C can remain in the plasma for a long period as a water-soluble vitamin. Thus, further explored whether vitamin C can affect RA progression via acting on plasma proteins associated with RA. MR findings identified 24 plasma proteins, which from 4907 plasma proteins published by Ferkingstad E et al,^[20]^ may induce RA. We observed that only RDH16 and ACP1 were the target proteins affected by vitamin C in terms of RA occurrence via the intersection between RA-associated plasma proteins and vitamin C targets. However, the potential mechanisms of action of these proteins in RA are still unclear. MR, which leverages genetic variants to explore potential causal relationships from a genetic perspective, revealed no significant association between genetically predicted vitamin C levels and RA risk. This suggests that the observed epidemiological link may be influenced by confounding factors rather than reflecting a direct protective effect.

Due to the inherent limitations of cross-sectional designs, including the inability to establish temporality and control for unmeasured confounders, these results should be interpreted with caution. Further validation through well-designed prospective cohort studies or randomized controlled trials is warranted to clarify the relationship between vitamin C intake and RA risk.

In summary, epidemiological studies from the NHANES database 2009 to 2018 suggest that vitamin C intake above 60 mg/day may the reduce risk of RA occurrence. However, the findings obtained through MR indicate that vitamin C intake may cause no effect the occurrence of RA from a biological perspective.

This study draws upon data from the 2009 to 2018 NHANES, a large, nationally representative cohort that enhances the external validity and generalizability of the findings. By combining conventional epidemiological methods with MR, we capture both observational associations and potential causal inferences between vitamin C intake and the risk of RA, thereby strengthening the overall analytical framework. Moreover, the investigation of dose-dependent effects and the incorporation of gut microbiota-vitamin C interactions provide a biologically plausible mechanism to support the hypothesized role of the gut joint axis in RA pathogenesis. The inclusion of sex-stratified analyses further refined the interpretation by accounting for well-documented immunomodulatory differences mediated by sex hormones in autoimmune diseases.

Despite these strengths, several limitations warrant consideration. First, dietary vitamin C intake was assessed using 24-hour dietary recall interviews, which are inherently prone to recall bias and may not accurately reflect long-term nutritional patterns. Second, although MR reduces confounding and reverse causality, the current paucity of genome-wide significant variants associated with circulating vitamin C levels may limit the statistical power and precision of the causal estimates. Third, while the study posits a potential microbiota-mediated pathway for vitamin C degradation in RA, this hypothesis remains speculative in the absence of direct experimental validation. In addition, potential confounding factors, such as concurrent intake of other micronutrients, pharmacological interventions, and underlying comorbidities, were not fully adjusted for and may influence the observed associations. Lastly, the lack of randomized controlled trials (RCTs) evaluating the impact of vitamin C intake within tolerable upper intake levels (UL) on RA incidence highlights the need for future longitudinal and interventional studies to confirm these findings and clarify underlying biological mechanisms.

## Acknowledgments

We thank all the NHANES work team and the providers of the public databases contributing to this study.

## Author contributions

**Data curation:** Ke Han.

**Visualization:** Ke Han.

**Software:** Shuqi Yuan.

**Methodology:** Suying Yan, Jiayu Fu.

**Validation:** Suying Yan.

**Formal analysis:** Tianyu Wu.

**Resources:** Tianyu Wu.

**Supervision:** Fangqin Cui, Xiaonan Zhang.

**Conceptualization:** Xiaonan Zhang.

**Project administration:** Xiaonan Zhang.

**Writing – review & editing:** Xiaonan Zhang.

**Writing – original draft:** Ke Han, Tianyu Wu.


